# 

**Published:** 1861-02

**Authors:** 


### CHICAGO MEDICAL JOURNAL.

**Terms, $2.00 per Annum.**

### CONTENTS OF THE FEBRUARY NUMBER.

ORIGINAL COMMUNICATIONS.

PAGE.

Observations on the Therapeutic Action of the Oxalate of Cerium in the Vomiting of Pregnancy. By H. W. Jones, A. M., M. D............ 65

Case of Diabetes Mellitus, in an advanced stage, Treated. By J. W. McKinney, M. D., of Camargo, Ill................................ 70

Cases of Gun Shot Wounds in the Right Lung, and their Treatment. By H. C. Clapp, M. D., Chicago, Ill................................ 73

A Case of Enteric Fever, with Remarks. By G. V. Ewing, M. D., Rock Run, Ill................................................ 81

SELECTED.

Treatment of Diphtheria. By Wm. Henry Thayer, M. D................ 86

" " (Local). By J. Kneeland, M. D................ 97

" " By A. Jacobi, M. D......................... 99

On Ice-Water in the Treatment of Croup. By J. A. McFarland, M.D., Tiffin, 100

On the Climate of California in its Relation to the Treatment of Pulmonary Consumption. By James Blake, M. D., F. R. C. S................. 102

PROCEEDINGS OF SOCIETIES.

Abstract of the Proceedings of the Esculapian Society. Reported by D. W. Stormont, M. D., Secretary................................ 107

The New Sydenham Society.............................................. 110

Chicago Academy of Medical Sciences.................................. 113

EDITORIAL.

Editorial and Miscellaneous........................................... 115

Rush Medical College................................................... 128

